# Factors associated with receipt of adjuvant chemotherapy among married women with breast cancer

**DOI:** 10.1186/1477-7819-11-286

**Published:** 2013-10-31

**Authors:** Yan Zhang, Hua Gao, Yulan Bu, Xiuzhen Fan, Jihui Jia

**Affiliations:** 1 Department of Nursing, Qilu Hospital and School of Nursing, Shandong University, West Wenhua Road, Ji'nan, Shandong 250012, P.R China; 2 Tianchen Hospital of Zaozhuang Mining Group, Tianchen Town, Zaozhuang City 277500, P.R China

**Keywords:** Breast cancer, Adjuvant chemotherapy, Epidemiology, Neoadjuvant chemotherapy

## Abstract

**Background:**

Adjuvant chemotherapies are recommended for most women after breast cancer surgery, and can greatly affect the patients’ survival. We describe and evaluate possible factors influencing receipt of postoperative adjuvant chemotherapy among breast cancer patients in China.

**Methods:**

A total of 1,431 women diagnosed with breast cancer from 1997 to 2005 were enrolled. We reviewed medical records and abstracted information about these patients. Details on social-demographic factors and clinical-pathological characteristics of participants were collected and analyzed. To meet our objectives, the patient’s age at diagnosis, comorbidities, menstrual status, rural/urban status, tumor size, lymph node status, distant metastasis, tumor stage and hormone receptor status were estimated.

**Results:**

Overall, 936 of these 1,431 patients (65.41%) received adjuvant chemotherapy. Receipt of chemotherapy was significantly associated with age at diagnosis, rural–urban disparities, and lymph node status of patients, though no significant difference was found between the age <50 and age 50 to 64 groups. Moderate association was also observed between hormone receptor status and receipt of adjuvant chemotherapy, though it was still not statistically significant.

**Conclusions:**

Our study suggests that age at diagnosis, rural–urban disparities and lymph node status of breast cancer patients are independent predictors for receipt of adjuvant chemotherapy among married Chinese women. Further investigations are warranted, and related public health education needs to be expanded in China.

## Background

Breast cancer has become the leading cause of cancer death among women in developing countries (for example, China), as well as the most diagnosed malignant tumor [1]. There are multiple strategies for breast cancer treatment, and adjuvant chemotherapy shows great benefit in reducing disease recurrence and improving prognosis for postoperative breast cancer patients [2,3]. Adjuvant chemotherapy is given after initial treatment with surgery, and is considered standard treatment for breast cancer patients [4-6]. Several studies have revealed that race, as well as marital status, may impact the receipt or completion of adjuvant chemotherapy among breast cancer patients [7-9]. We describe and evaluate possible factors influencing receipt of postoperative adjuvant chemotherapy among married yellow-race women with breast cancer in China.

## Methods

### Patients and data collection

Females diagnosed with primary breast cancer between 1 January 1 1997 and 31 December 2005 and who received surgery treatment post-diagnosis in Qilu Hospital were collected as candidates. All cases are yellow race, married females who met clinical criteria for consideration of adjuvant chemotherapy. We retrieved and identified information from medical records. To meet our objectives, social-demographic factors and clinical-pathological characteristics of candidates including patient’s age at diagnosis, comorbidities, menstrual status, rural/urban status, tumor size, lymph node status, distant metastasis, tumor stage, hormone receptor status, race, marital status, and others were abstracted.

Breast cancer cases were identified by operation and histopathological examination. Clinical-pathological parameters such as estrogen receptor (ER) and progestogen receptor (PR) were evaluated by immunological histological chemistry staining. Cases were scored positive when ≥10% of the tumor cells on the slide showed positive staining irrespective of staining intensity. Operating procedures were classified into radical mastectomy, modified radical mastectomy and breast/nipple conserving surgery. Rurality was defined according to the socioeconomic survey team of the National Bureau of Statistics of China [10]. Comorbid illness includes cardiovascular diseases, rheumatism, tuberculosis, liver disease and other chronic diseases excluding previous or current malignant tumors that were reported by patients or their relatives. Patients without menses for more than six months were classified as postmenopausal cases. All variables were measured as of the time of treatment. Studies have shown that receipt of neoadjuvant chemotherapy may influence decisions about adjuvant chemotherapy [7,11,12]. As a result, whether a patient had received neoadjuvant chemotherapy was identified and classified.

Data collection was performed independently by two individuals. Patients’ information was abstracted and coded anonymously for all participants in the study. Written, informed consent was obtained as delineated by the protocol, which was approved by the ethical committee of Shandong University.

### Statistical analyses

We conducted univariate analyses with each of these explanatory variables using Chi-square (*χ*^*2*^) tests accompanied by two-sided *P* values. A multivariate logistic regression model evaluated the association between predictors and receipt of adjuvant chemotherapy. Because the factors that affect delivery of neoadjuvant chemotherapy are probably similar to those that affect adjuvant chemotherapy, we removed parameters of neoadjuvant chemotherapy from the multivariate analyses. Logistic regression outcomes were expressed as adjusted odds ratios (OR), with 95% confidence intervals (CI). For estrogen receptor/progestogen receptor (ER/PR) status, the predictor variable showed a significant number of missing values; therefore, we re-estimated the relevant model as a sensitivity test. All the statistics were performed using the PASW Statistics 18.0 software (SPSS Inc. Chicago, Illinois, USA), and were based on two-tailed probability. *P* values of <0.05 were considered statistically significant.

## Results

During the period from 1 January 1997 to 31 December 2005, a total of 1,431 married women who were diagnosed with invasive breast cancer (pTNM stage I - IV) and who received breast surgery in Qilu Hospital was included in our analyses. Overall, 936 of these 1,431 patients (65.41%) received adjuvant chemotherapy, and patients’ social-demographic and clinical-pathological characteristics were described in Table 1. We included possible predictors into our univariate analyses, as shown in Table 2. The percentage of rural women who received chemotherapies was significantly lower when compared with urban females (61.15% versus 67.38%, *P* = 0.021). Meanwhile, our results showed that receipt of chemotherapy was significantly associated with patient’s age at diagnosis (*P* = 0.030), with older females receiving less adjuvant chemotherapy. Whether a patient received neoadjuvant chemotherapy was related to the final decision of receipt of adjuvant chemotherapy or not (*P* <0.001). Importantly, outcomes demonstrated that lymph node status of patients was highly associated with their final decision about receipt of adjuvant chemotherapy (*P* <0.001). In these unadjusted analyses, no significant association was found between comorbid status, menopausal status, tumor size, distant metastasis or tumor stage and receipt of chemotherapy. Moderate association was observed between hormone receptor status and receipt of chemotherapy, with a *P* value of 0.082.

**Table 1:** Demographic and clinical characteristics of breast cancer patients

**Variable**	**Number of cases (N = 1,431)**
Age at diagnosis (years)	
<50	780
50 to 69	572
≥70	79
Neoadjuvant chemotherapy received	
No	1,075
Yes	356
Comorbidities	
None	1,024
One or more	407
Menopausal status	
Premenopause	703
Menopause	728
Residential status	
Rural	453
Urban	978
Smoking	
Previous or current	13
Never or no report	1,418
Reasons for visit	
Self-examination	1,404
Physical examination	27
Tumor size	
≤2	1,088
>2	214
Lymph node metastasis	
Negative	823
Positive	606
Distant metastasis	
Negative	1,421
Positive	10
Tumor stage	
I	178
II	927
III	198
IV	10
Hormone receptor status	
ER- and PR-	203
ER + and/or PR+	648
Histological type	
IDC	1,164
ILC	49
Others	218
Procedure	
Radical mastectomy	503
Modified radical mastectomy	828
Breast/nipple conserving surgery	55
NOS	45
Treatment	
Surgery	1,431
Radiotherapy	11
Endocrinotherapy	264
Chemotherapy	936
NOS	496

ER, estrogen receptor; PR, progestogen receptor; IDC, invasive ductal carcinoma; ILC, invasive lobular carcinoma; NOS, not otherwise specified.

**Table 2:** Univariate analysis of demographic and clinical factors associated with receipt of adjuvant chemotherapy

**Variable**	**Number of cases ** **(N = 1,431)**	**Percent that received adjuvant ** **chemotherapy (N = 936)**	***χ***^***2 ***^ ***P***
Age at diagnosis (years)			
<50	780	79.62%	0.030
50 to 69	572	65.38%	
≥70	79	51.90%	
Neoadjuvant chemotherapy received			
No	1,075	60.28%	<0.001
Yes	356	80.90%	
Comorbidities			
None	1,024	65.43%	0.979
One or more	407	65.36%	
Menopausal status			
Premenopause	703	66.29%	0.492
Menopause	728	64.56%	
Residential status			
Rural	453	61.15%	0.021
Urban	978	67.38%	
Tumor size			
≤2	1,088	67.2%	0.369
>2	214	64.0%	
Lymph node metastasis			
Negative	823	61.6%	<0.001
Positive	606	70.6%	
Distant metastasis			
Negative	1,421	65.4%	0.759
Positive	10	70.0%	
Tumor stage			
I	178	64.0%	0.614
II	927	67.6%	
III	198	63.6%	
IV	10	70.0%	
Hormone receptor status			
ER- and PR-	203	61.58%	0.082
ER + and/or PR+	648	71.91%	

ER, estrogen receptor; PR, progestogen receptor.

Then we estimated the adjusted values by controlling covariates using multivariate logistic regression model, as shown in Table 3. Interestingly, the statistical pattern of result did not change, and the significant association demonstrated in the univariate analyses still existed in the multivariate analyses, with the exception of neoadjuvant status, a parameter that had been removed from the analytic models. Receipt of chemotherapy was significantly associated with age at diagnosis, rural–urban disparities, and lymph node status of patients. The difference between age <50 and age ≥70 was significant (OR = 0.407, 95% CI = 0.209 to 0.792, *P* =0.008), though no significant difference was found between age <50 and age 50 to 64 groups (*P* = 0.319). More urban females received adjuvant chemotherapy than rural ones after adjusting r other parameters (OR = 1.381, 95% CI = 1.083 to 1.761, *P* = 0.009). Patients with positive lymph node metastases received adjuvant chemotherapy more often than patients with negative cases of lymph node metastases (OR = 1.600, 95% CI = 1.266 to 2.022, *P* <0.001). Moderate association was also observed between hormone receptor status and receipt of adjuvant chemotherapy, though it was still not statistically significant (*P* = 0.066). Because ER/PR status is an important pathological factor and because observations on this variable were missing for 38.71% of the cases, we performed a sensitivity analysis to verify our result (not shown). We re-estimated the logistic regression model (1) by excluding hormone receptor (ER/PR) status or (2) by only accepting the remaining 887 patients with ER/PR information. No change in sign or patterns of statistical significance was found for any of them.

**Table 3:** Logistic regression analysis of demographic and clinical factors associated with receipt of adjuvant chemotherapy

**Variable**	**OR**^**a**^	**95% CI**^**a**^	**Adjusted *P***^**a**^
Age at diagnosis (years)			
<50	Referent		
50 to 69	0.769	0.463 to 1.275	0.308
≥70	0.410	0.212 to 0.793	0.008
Comorbidities			
None	Referent		
One or more	1.033	0.791 to 1.349	0.811
Menopausal status			
Premenopause	Referent		
Menopause	1.205	0.734 to 1.979	0.461
Residential status			
Rural	Referent		
Urban	1.381	1.083 to 1.761	0.009
Tumor size			
≤2	Referent		
>2	0.828	0.603 to 1.136	0.242
Lymph node metastasis			
Negative	Referent		
Positive	1.600	1.266 to 2.022	<0.001
Distant metastasis			
Negative	Referent		
Positive	1.123	0.282 to 4.475	0.869
Hormone receptor status			
ER- and PR-	Referent		
ER + and/or PR+	1.364	0.979 to 1.900	0.066

^a^Adjusted using multivariate logistic regression model.

ER, estrogen receptor; PR, progestogen receptor; OR, odds ratios; CI, confidence intervals.

Previous studies have mentioned that receipt of neoadjuvant chemotherapy could influence a patient’s decisions about receipt of adjuvant chemotherapy [7,11,12]. Consequently, we excluded patients who had received neoadjuvant chemotherapy (as have other studies mentioned above) and re-estimated the logistic regression model in Table 4. Overall, 1,075 patients were included, and there were no changes in sign or patterns of statistical significance for any of the parameters. Because the factors that affect delivery of neoadjuvant chemotherapy are probably similar to those that affect adjuvant chemotherapy, and because women get all of their chemotherapy as neoadjuvant in some countries, we also re-estimate our analytic model by combining neoadjuvant and/or adjuvant chemotherapy as dependent variables, as shown in Table 5. A moderately significant association was observed between rural–urban disparities and receipt of adjuvant chemotherapy, with a *P* value of 0.072. No changes in sign or patterns of statistical significance were observed for the remaining factors.

**Table 4:** Logistic regression analysis of factors associated with receipt of adjuvant chemotherapy among patients not receiving neoadjuvant chemotherapy

**Variable**	**OR**^**a**^	**95% CI**^**a**^	**Adjusted *P***^**a**^
Age at diagnosis (years)			
<50	Referent		
50 to 69	0.690	0.386 to 1.234	0.211
≥70	0.303	0.143 to 0.643	0.002
Comorbidities			
None	Referent		
One or more	1.090	0.810 to 1.467	0.568
Menopausal status			
Premenopause	Referent		
Menopause	1.374	0.778 to 2.428	0.274
Residential status			
Rural	Referent		
Urban	1.398	1.058 to 1.846	0.018
Tumor size			
≤2	Referent		
>2	0.799	0.510 to 1.150	0.198
Lymph node metastasis			
Negative	Referent		
Positive	1.383	1.057 to 1.809	0.018
Distant metastasis			
Negative	Referent		
Positive	1.908	0.191 to 19.015	0.582
Hormone receptor status^a^			
ER- and PR-	Referent		
ER + and/or PR+	1.425	0.981 to 2.070	0.063

^a^Adjusted using multivariate logistic regression model.

ER, estrogen receptor; PR, progestogen receptor; OR, odds ratios; CI, confidence intervals.

**Table 5:** **Logistic regression analysis of demographic and clinical factors associated with receipt of adjuvant and**/**or neoadjuvant chemotherapy**

**Variable**	**OR**^**a**^	**95% CI**^**a**^	**Adjusted *P***^**a**^
Age at diagnosis (years)			
<50	Referent		
50 to 69	0.701	0.407 to 1.207	0.200
≥70	0.337	0.169 to 0.674	0.002
Comorbidities			
None	Referent		
One or more	1.075	0.813 to 1.420	0.613
Menopausal status			
Premenopause	Referent		
Menopause	1.319	0.773 to 2.249	0.310
Residential status			
Rural	Referent		
Urban	1.265	0.979 to 1.633	0.072
Tumor size			
≤2	Referent		
>2	1.266	0.888 to 1.804	0.192
Lymph node metastasis			
Negative	Referent		
Positive	1.933	1.508 to 2.477	<0.001
Distant metastasis			
Negative	Referent		
Positive	2.639	0.326 to 21.335	0.363
Hormone-receptor status			
ER- and PR-	Referent		
ER + and/or PR+	1.392	0.986 to 1.964	0.060

^a^Adjusted using multivariate logistic regression model.

ER, estrogen receptor; PR, progestogen receptor; OR, odds ratios; CI, confidence intervals.

All the logistic regression models adopted in this study exhibited high goodness-of-fit, with a Hosmer-Lemeshow goodness-of-fit *P* value of 0.996 in Table 3 and above 0.500 in all logistic models.

## Discussion

Receipt of proper adjuvant chemotherapy for breast cancer patients is very important and associated with prognosis of breast cancer patients. Both clinical trials and consensus guidelines have identified subsets of women with breast cancer who are able to gain benefit from adjuvant chemotherapy [4,13-15]. Many factors, including age, comorbidities, marital status, variation in provider recommendation, medical insurance, and life expectancy, are associated with whether women receive adjuvant chemotherapy and have been reported for a long time [12,16-20]. Married women usually enjoy overall better health and higher socioeconomic status than unmarried ones, which may translate into better access to healthcare and other benefits. Meanwhile, marriage may reflect a healthy selection bias, and one study has reported that those with psychiatric or physical impairments may be less likely to marry [21]. Marriage may also influence the lifestyle and behaviors of women, including diet, exercise, and health screening, which may be mediating factors when making better medical choices [22]. On the other hand, marriage could offer better social support networks [23], indicating better financial support and receipt of more sensible advice, which could influence a woman’s choice about receipt of adjuvant chemotherapy. Considering that most of our candidates were married, this study only focused on married women to reduce bias resulting from these confounding factors. Our research focused on married Chinese women who met clinical criteria for adjuvant chemotherapy, and analyzed the possible predictors about receipt of adjuvant chemotherapy. Though the recommendations and protocols for adjuvant chemotherapy changed over this period, an inpatient who was recommended for chemotherapy but refused doctors’ advice would be recorded for the purpose of preventing medical accidents, so we were able to select candidates for this study. For now, only patients with invasive breast cancers are recommended for adjuvant chemotherapy generally, and we performed secondary selection only accepting patients diagnosed with invasive breast cancers, in line with current research. Because health care in China is a government-run project and most of our patients on record were covered by this basic medical insurance system, we did not take individual financial income of breast cancer patients into consideration.

In the univariate analyses, we found that 65.41% of the patients in this research have received postoperative adjuvant chemotherapy. Age at diagnosis, rural–urban disparities, and receipt of neoadjuvant chemotherapy or not, significantly associated with the final decision of patients about the receipt of adjuvant chemotherapy, as shown in Table 2. After being controlled for covariates, the differences still existed and no change in sign or patterns of statistical significance was observed in all participants and cases without receipt of neoadjuvant chemotherapy only, as shown in Table 3 and Table 4, respectively.

Age of patients is associated with receipt of adjuvant chemotherapy and has been studied for a long time [3,11,16,24-26]. Our data revealed that older women adopt adjuvant chemotherapy less than young women, especially women at least 70 years old. In the clinic, older patients are less likely to tolerate chemotherapy and more likely to adopt more conservative treatment for a better quality of remaining life, consistent with clinical reports and consensus guidelines [14,24,27]. We also found that more urban women adopted adjuvant chemotherapy than rural ones, and the difference is statistically significant. Rural–urban disparities have an influence on many aspects for Chinese females, especially on education, life style, income and so forth, and urban women usually receive a better education and make more money. All of these could result in urban women knowing the benefit of adjuvant chemotherapy better, being more willing and able to pay for their health, and being able to endure the pain and chemotherapy.

As mentioned in previous studies, receipt of neoadjuvant chemotherapy may influence the decisions about adjuvant chemotherapy [7,11]. Our results indicate that patients who received neoadjuvant chemotherapy are more likely to receive postoperative chemotherapy (80.90% versus 60.28%, *P* <0.001). Because the factors that affect delivery of neoadjuvant chemotherapy are probably similar to those that affect adjuvant chemotherapy, we re-estimated the predictors among females without receipt of neoadjuvant chemotherapy or combined neoadjuvant and/or adjuvant chemotherapy as one dependent variable. After that we still had similar results, which verify the predictors discovered in our analyses.

### Strengths and weaknesses

First, chemotherapy regimens adopted by patients in our study consist of CMF, CTX, CEF and so forth, and we did not include chemotherapy regimens as parameters in our analyses. However, it would make our analytic models overly complicated and affect the accuracy and stability of our results since many patients accepted chemotherapy with multiple regiments. Second, a significant number of predictor values (ER and PR status) were missing in the multivariate analytic models. Nonetheless, we re-estimated the relevant model by excluding hormone-receptor (ER/PR) status or by only accepting the remaining 887 patients with ER/PR information in the analyses, and found no change in sign or patterns of statistical significance for any of them.

## Conclusions

Recently the importance of understanding patients who did or did not receive advised adjuvant chemotherapy was highlighted and discussed [3,7,8]. Studies have discovered that whether a patient who met clinical criteria for adjuvant chemotherapy accepted it or not, is associated with human race, marital status, socioeconomic status, and some other demographic factors. In this study, we collected information on female breast cancer patients who are yellow race and married, and explored the predictors about receipt of adjuvant chemotherapy. Our study suggests that age at diagnosis, rural–urban disparities and lymph node status of breast cancer patients are independent predictors for the receipt of adjuvant chemotherapy among married Chinese women. Further investigations are warranted, and related public health education needs to be expanded in China.

## Abbreviations

CI: Confidence interval; ER: Estrogen receptors; IDC: Invasive ductal carcinoma; ILC: Invasive lobular carcinoma; NOS: Not otherwise specified; OR: Odds ration; PR: Progestogen receptor.

## Competing interests

The authors declare that they have no competing interests.

## Authors’ contributions

YZ, XF and JJ conceived of the study and drafted and revised the manuscript. YZ, HG and YB carried out the data collection and the statistical analysis. All authors read and approved the final manuscript.
